# Psychometric Evaluation of the Chinese Version of the Patient Perceptions of Empowerment Scale (PPES)

**DOI:** 10.1155/2014/867451

**Published:** 2014-05-29

**Authors:** Mei-Yu Yeh, Sieh-Hwa Lin, Tao-Hsin Tung

**Affiliations:** ^1^Graduate Institute of Health Care, Chang Gung University of Science and Technology, Taoyuan 33303, Taiwan; ^2^Department of Educational Psychology and Counseling, National Taiwan Normal University, Taipei 10610, Taiwan; ^3^Department of Public Health, School of Medicine, Fu-Jen Catholic University, Taipei 24205, Taiwan; ^4^Department of Medical Research and Education, Cheng-Hsin General Hospital, Taipei 11220, Taiwan

## Abstract

*Objectives*. To evaluate the psychometric properties of the Chinese version of the Patient Perceptions of Empowerment Scale (PPES) and to perform a cross-cultural validity assessment. *Methods*. In this cross-sectional survey, 554 inpatients in three general hospitals in northern Taiwan were recruited. Principal component analysis was used to examine the factor structure of the scale. Confirmatory factor analyses were conducted on the measurement model of the Chinese version of the PPES. *Results*. Confirmatory factor analyses supported the presence of a second-order four-factor model (information, decision, individual, and self-management) of the Chinese version of the PPES when used with a Taiwanese inpatient population. The results indicate that the 11-item, second-order, four-factor Chinese version of the PPES provided best goodness-of-fit for the data in this study. *Conclusion*. The 11-item four-factor Chinese version of the PPES is a self-completion scale. This study demonstrated that the Chinese version of the PPES is a reliable and valid self-report instrument for the assessment of patient perceptions of empowerment in clinical practice. Further adaptation and evaluation of the scale will hopefully stimulate further studies on PPES in the fields of psychometrics in Taiwan.

## 1. Introduction

The term “empowerment” comes from the Latin verb for power, “potere,” which means “to be able.” Its prefix, “em,” means “cause to be” or “provide with” [1]. The Oxford English Dictionary defines empowerment as “to give someone the power to do something; to make someone more confident, especially in controlling their life and claiming rights” [2]. Hence, empowerment means to give power to (empower) or to make able (enable) [3].

From the evidence-based viewpoint, empowerment is a process of awareness, enabling involved individuals to assert control over the problems that affect their health, thus achieving self-determination. Within an empowerment framework, the responsibility of health-care providers is to recognize the suffering of patients, identify patients' strengths, and prevent further marginalization of patients due to power inequality [4, 5]. In the empowerment process, the patient has the right to choose their treatment. Health care providers provide clear, concise, accurate information [6] and patients can access and discuss a range of information, options, and views to help them self-determine and self-manage their disease [3, 7]. The concept of patient empowerment emphasizes health care to be patient-centered, providing educational materials and decision-making aids, such as leaflets, computer programs, interactive videos, websites, and group presentations [8]. These materials help patients identify their own skills and needs and empower themselves instead of just focusing on compliance with the offered treatments [7, 8].

The concept of patient empowerment is complex and multidimensional and is a construct applicable to individuals, communities, and organizations [9, 10].

The Patient Perceptions of Empowerment Scale (PPES) was used to investigate coronary-care inpatients' perceptions of interventions related to patient choice, and to identify the patient empowerment benchmarks within clinical practice. It also was linked to concepts of self-care, personal control, and demonstrating the ability, confidence, and insight to enhance the individual's well-being [11]. In addition, the psychometric properties of PPES includes 5 factors: giving of information about treatment and care, having a voice in the choice and direction of clinical management, getting an informed consent by the patient prior to treatment, providing individualized care, respecting the patient as an individual, and self-management of diseases with knowledge and confidence [11]. Although a number of empowerment (and other) scales are presented (Table 1) [11–19], only PPES could be used in clinical research to predict inpatient empowerment and to evaluate patient-education outcomes. The objective of this study was to examine the psychometric properties of the Chinese version of the PPES and to perform a cross-cultural validity assessment.

## 2. Methods

### 2.1. Participants

The survey was conducted in three general hospitals in Taiwan from January to July 2009. We excluded patients in pediatrics, psychiatry, and intensive care for difficulty of collecting accurate data. We recruited 554 patients who were able to express willingness in either Mandarin or Taiwanese and who had been hospitalized for three or more days in internal medicine, surgery, gynecology, neurology, or one or more of the 18 other wards. A total of 554 inpatients met these inclusion criteria and comprised the study sample. All participants completed a questionnaire pack at one time point.

### 2.2. Ethical Considerations and Procedures

The institutional review boards at three hospitals approved this study in 2008. The research procedures and recruiting criteria were explained to nurses before they contacted potential subjects. All participants were referred by nurses and gave informed consent for participation in this study. It was emphasized that participation was voluntary and could be withdrawn at any time, and subjects' responses were to be considered anonymous and confidential. The researchers explained the risks and benefits of participation, and the patients' right to refuse to participate without jeopardizing treatment. Participants were given verbal and written explanations of the study's purpose and design. Methods and procedures of data collection, use and analysis, and storage were explained. Research data was then collected using a self-report questionnaire, in which the patients were required to complete within 20 minutes.

### 2.3. Instruments

#### 2.3.1. The Original Patient Perceptions of Empowerment Scale (PPES)

The original version of the PPES contains 17 items and uses a five-point Likert scale, which ranged from strongly agree to strongly disagree. The factors of the PPES are as follows: giving of information about treatment and care; having a voice in the choice and direction of clinical management; getting an informed consent by the patient prior to treatment; providing individualized care and respecting the patient as an individual; and self-management of diseases with knowledge and confidence [11].

The Chinese version of the PPES was created using the back-translation method. First, the original version was translated into Chinese by a professional translator. The first author then back-translated the Chinese version, and the result was compared with the original English version to ensure that the words chosen in the translation carried their original connotations.

Content validity is used to assess the relevance and the comprehensiveness of the items. Before conducting our preliminary research, five nursing experts based on their clinical professions and experiences examined the content validity; that is, when experts were asked whether they missed items this could be considered as an indication that the comprehensiveness of the items was assessed. The five experts included a clinical specialist, two nursing supervisors, and two assistant professors. In order to avoid that the particular composition of participants may bias towards certain subdimensions of the scale, convergent validity and discriminant validity are assessed through confirmatory factor analyses (CFA). In addition, the information regarding the construct reliability (composite reliability, a measure of the overall reliability of a collection of heterogeneous but similar items) and construct validity (the degree to which the performance of the items on a translated or culturally adapted PPES instrument is adequate reflection of the performance of the items of the original version of the PPES instrument) of the scale was considered to test the psychometric properties of the PPES in this study.

#### 2.3.2. The Patient Satisfaction Questionnaire (PSQ)

The PSQ has frequently been used as a measurement of quality of nursing care, especially in attempts to demonstrate the patients' experiences during and post of treatment. The PSQ includes 11 questions on a Likert scale [20]. The PSQ range of total scores is 11–55, with higher scores indicating higher degrees of satisfaction with nursing care. The internal consistency of the scale was estimated using Cronbach's alpha coefficients. The coefficients of internal consistency greater than 0.80 or 0.90 are considered as very good or excellent [21]. In our pilot study, the PSQ total-scale Cronbach's alpha coefficient was 0.91.

#### 2.3.3. Sufficiency of Patient Education Questionnaire (SPEQ)

The SPEQ has been used to evaluate whether the education patients received was sufficient within the hospitalization period. The SPEQ includes 8 items on a Likert scale. The range of total scores is 8–40, with lower scores indicating less-sufficient patient education within the hospitalization period [22]. In our pilot study, the Cronbach's alpha coefficient of SPEQ was 0.92.

### 2.4. Statistical Analysis

SPSS version 20.0 for Windows was used to perform descriptive statistics, Pearson correlation, and exploratory factor analysis (EFA). The dimensionality of the scale was evaluated by EFA, using Kaiser's criterion (eigenvalue ≥1), estimated by the maximum-likelihood method and rotated with the varimax method [21]. CFA were also conducted using the second-order four-factor measurement model of the PPES based on results of EFA. These analyses were performed using the structural-equation modeling (SEM) statistics package LISREL 8.80.

The goodness-of-fit of the models was estimated using the covariance matrix as input and a maximum-likelihood solution [23, 24]. The LISREL software program was used to examine the parameter, test the hypothesis model, and estimate the population covariance matrix generated by this model [25, 26]. Browne and Cudeck [27] indicated that there were multiple goodness-of-fit indices to provide information on how well the model fits the data. Using structural-equation modeling, several model-fit indices were evaluated: the Root Mean Square Error of Approximation (RMSEA), Incremental Fit Index (IFI), Comparative Fit Index (CFI), Nonnormed Fit Index (NNFI), Goodness of Fit Index (GFI), and Standardized Root Mean Square Residual (SRMR). RMSEA values smaller than 0.10 indicate a good fit, with values below 0.05 indicating a very good fit. CFI, GFI, IFI, and NNFI values greater than 0.90 indicate a good-fit [24, 25, 28]. SRMR values less than 0.05 are interpreted as indicating a good fit to the data [23]. In addition, construct validity was used to explore the expected correlations between PPES and PSQ and between PPES and SPEQ. The construct validity of the PPES was assessed by second-order factor analyses [29]. The purpose of the second-order factor analysis is to explore how strongly the first-order factors load on the hypothesized second-order factor, that is, to estimate to what extent the explained factors of meaning, competence, self-determination, and impact could be accounted for by the more generic concept of PPSE [21]. The second-order factor analysis also could be used to study construct validity because it assesses the degree to which some items load on a certain hypothesized first-order factor and at the same time load very insignificantly on other first-order factors [21]. Finally, we examined raw scores and Pearson's correlation coefficients; the correlations were performed using the SPEQ and PSQ to assess the concurrent validity.

## 3. Results

The content validity index (CVI) for the PPES was 0.93. As recommended by these experts; we removed two questions which did not reflect clinical practice in Taiwanese hospitals; the 9th and 13th items were removed. The second draft of the PPES, at this point containing 15 items, was evaluated for item clarity by 20 inpatients. The 8th and 16th items were regarded as socially and culturally inapplicable and, therefore, deleted, leaving 13 items.

In the pilot study, we collected data from 30 inpatients to examine internal consistency. The 13-item PPES total-scale reliability coefficient was 0.86. Item analyses were then used to examine how each item correlated with the total score. Items with item-to-total correlations lower than 0.3 were deleted, because they did not sufficiently contribute to measuring the concept and reduced the scale's homogeneity [20]. At this stage, we deleted the 4th (*r* = −0.15) and 5th (*r* = −0.08) items. The final 11-item version of the PPES was used for the rest of this study.

A total of 554 inpatients participated in this study, in which 291 (52.5%) were male and 263 (47.5%) were female. Ages of the participants ranged from 20–99 years (mean age = 52.75). Participants had an average of 9.94 years of education and 9.9% had not been employed recently.

Using principal component analysis, the researchers analyzed the construct validity of the Chinese version of the PPES by using varimax rotation to extract four factors (Table 2). The exploratory factor analysis was performed in order to be able to directly inspect whether or not the factor-loading matrix possessed the so-called simple structure. The four extracted factors explained 70.57% of the total variances. The factor loadings of the 11 questions were between 0.58 and 0.88, the Kaiser-Meyer-Olkin (KMO) measure of sampling adequacy was 0.888, and the Bartlett test of sphericity was 2484.266 (df = 55;  *P* < 0.001). The four factors were named “information” (three items), “decision” (two items), “individual” (three items), and “self-Management” (three items). The Cronbach's alpha values of the four subscales (four factors) were between 0.63 and 0.81.

All standardized factor loadings of items were significant (*t* value larger than 2), with factor loadings ranging from 0.68 to 0.84 for the information factor, 0.65 to 0.78 for the decision factor, 0.55 to 0.90 for the individual factor, and 0.55 to 0.71 for the self-management factor. Total cumulative variances ranged from 0.62 to 0.77 in the four-factors (Table 3). These results indicate good convergent validity [28, 30]. Bagozzi and Yi indicated that a construct reliability (composite reliability) higher than 0.60 and total cumulative variances greater than 0.50 are acceptable [31]. In the present study, the construct reliability was 0.93 for information, 0.80 for decision, 0.93 for individual, and 0.84 for self-management (Table 3). These findings support the second-order four-factor model proposed by exploratory factor analysis and the construct validity of the PPES.

The goodness-of-fit analysis revealed *χ*2 = 122.37,  df = 40,  *P* < 0.001,  NC = 3.05, CN = 281.15, RMSEA = 0.061,  IFI = 0.98,  CFI = 0.98,  NNFI = 0.98,  GFI = 0.96, and SRMR = 0.045. These results clearly indicate that the second-order four-factor model has a reasonable degree of approximation for this population and is acceptable for use in research and clinical applications (Table 4).

Significant correlation coefficients were 0.68 (*P* < 0.01) for the SPEQ and 0.64 (*P* < 0.01) for the PSQ, providing concurrent evidence for validity. The significant correlation coefficients for individual factors within SPEQ were 0.60 (*P* < 0.01) for information, 0.59 (*P* < 0.01) for decision, 0.55 (*P* < 0.01) for individual, and 0.47 (*P* < 0.01) for self-management (Table 4). These results indicate that the more sufficient patient education is, the more empowered the individual is likely to control or self-manage their problem. The correlation value was in the expected direction and supported the concurrent validity of the SPEQ and PSQ.

## 4. Discussion

### 4.1. Discussion

In the present study, the four-factor model of the Chinese version of the PPES was simplified to 11 items. Each factor has two to three questions in which adequate convergent validity is all embodied. Confirmatory factor analyses provided moderate support for the 11-item four-factor model of the PPES. Therefore, this second-order four-factor solution of the Chinese version of the PPES is a best-fit model verified for use in Taiwan [28, 30]. In terms of the concepts of PPES, four-factor was also related to perceived patient satisfaction and linked with the concepts of self-care, self-responsibility, and personal control [11]. Piper indicated the nurse as an empowerment facilitator that focuses on patient-defined needs, making sure patients are fully aware of treatment options and implications and fosters active patient participation [32]. Interventions are grounded in process and empathy, reassurance, and support helped to develop critical awareness, confidence, self-esteem, and thus bottom-up patient-led decision-making [33].

**Figure 1:**
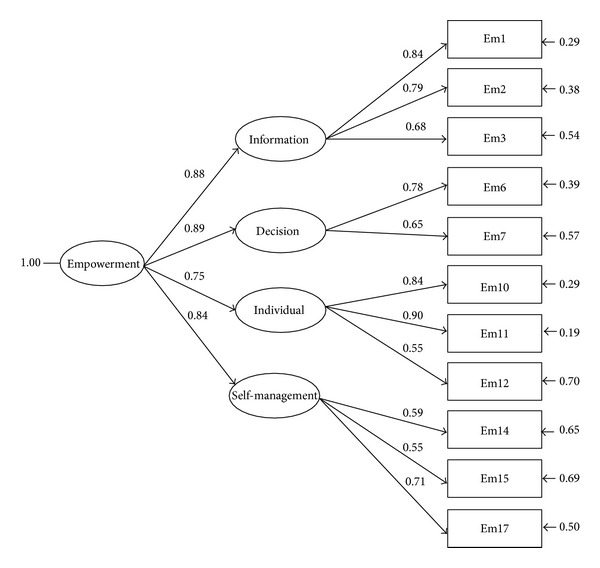
Second-order four-factor model of the PPES.

For all of the groups, the factor solutions brought about exactly four factors that justified over 70 percent of the total variance. Factor I (information) explicated most of the total variation. For both groups, the scale might be regarded as having construct validity. As Figure 1 points out, all second-order loadings (*y*) were high (ranging from 0.55 to 0.90) and all first-order loadings (*π*) were also high and statistically important (varying between 0.75 and 0.89). In addition, Kline reported that discriminant validity refers to the distinctiveness of the factors measured by different sets of indicators [28]. If the estimated correlations of the factors that underlie sets of indicators that are supposed to measure different constructs are not excessively high, then there is evidence for discriminant validity. Although the Pearson correlation coefficient associations were slightly and moderately significant amongst the SPEQ, PSQ, and second-order four-factor model of the PPES, the significance exists and is statistically meaningful [28, 30]. According to Kline, when correlations are examined and the *γ* value is moderately significant, it can be interpreted as concurrent validity [28]. Principal component analysis and confirmatory factor analysis of the data in this study confirmed second-order four factors within the Chinese version of the PPES.

### 4.2. Methodological Considerations

Several limitations should be considered when interpreting the results of this study of the Chinese version of the PPES. A very important limitation of this study is that neither stability (test retest reliability) nor responsiveness (sensitivity to change) of the Chinese PPES was assessed. Arguably, these are the key properties that would make a measure useful in the clinical context. Another major limitation to this study population is selected on a voluntary basis, which would potentially introduce selection bias. Voluntary bias can be defined as the result of the fact that a particular sample can contain only those participants who are actually willing to participate in the study and who participate and find the topic particularly interesting are more likely to volunteer for that study, same to those who are expected to be evaluated on a positive level [34]. Thirdly, this study does not present further evidence for the construct or discriminant validity of the scale. For example, the differences may occur in endorsing the underlying dimensions by participants of different age, gender, and patient characteristics. Fourthly, our measurements were conducted at only a single point in time and, by clear inference, would not only be able to be used to reflect long-term exposure to various aspects or factors, which might be important influencers of PPES, but also only internal consistency was assessed due to no demonstrated reliability of the Chinese PPES in terms of stability over time. Fifthly, published standards for translation of health measurement scales recommend 2 independent translations, review by expert panel, 2 independent back-tranlsations, and 2nd review by expert panel [35, 36]. However, due to limited resources, the original version was translated only by a professional translator. The measurement error was inevitable. In order to ensure that the translation procedures were robust, further studies should consider conducting some cognitive interviews to ensure the Chinese wording of the items was appropriate [37]. Finally, the PPES constructs were developed by scholars in the United Kingdom considering the characteristics of the specific population studied. While the PPES was modified to fit the population in this study, future cross-cultural studies may illuminate other culturally-sensitive issues.

### 4.3. Practice Implications

The Chinese version of the PPES has the potential to measure and evaluate quality of healthcare related to patient-empowerment concepts. It can be used to improve the outcomes of clinical-care services in Taiwan in relation to the level of perceptions of empowerment in clinical practice. Due to the fact that stability and responsiveness of the Chinese PPES are yet to be demonstrated, further adaptation and evaluation of the scale will hopefully stimulate further studies on PPES in the fields of psychometrics in Taiwan.

### 4.4. Conclusions

The 11-item four-factor Chinese version of the PPES is a self-completion scale. It includes the following factors: information (three items), decision (two items), individual (three items), and self-management (three items). This study demonstrated the Chinese version of the PPES to be a reliable and valid tool for both evaluating patient-empowerment outcomes and assessing patient-empowerment education in clinical and research practice.

## Figures and Tables

**Table 1:** Summarized research results of empowerment scale.

Author(s)	Measure	Methods	Subjects	Results
Lewin and Piper [11]	17-item patient perceptions of empowerment scale	Frequency score and rank	142 inpatients	The 17 items were rescored on a five-point scale; the higher score, the more satisfied the respondent

Anderson et al. [12, 13]	28-item diabetes-patient empowerment scale	Principal component analysis	375 and 229 diabetes patients	Three-factor solution accounts for 56% of the total variance

Bulsara et al. [14]	28-item patient empowerment scale	Rasch model analysis	100 cancer patients	Fitted the Rasch model with the exception of 2 items

Faulkner [15]	100-item patient- empowerment/disempowerment scale (frequency-of- action scale)	Frequency score	102 elderly patients	Offered as a means of identifying hospital environments which facilitate independence

Chen et al. [16]	15-item diabetes-patient empowerment process scale	Principal component- and confirmatory factor analysis	211 diabetes patients	Second-order four-factor model; four factors: raising awareness, mutual participation, providing information, and communication

Hansson and Björkman [17]	28-item empowerment scale	Confirmatory factor analysis	176 subjects with mental illness	Good construct validity; two-factors: self-esteem and activism and community and power

Kettunen et al. [18]	43-item empowering-speech scale	Confirmatory factor analysis	127 counseling situations	Second-order two-factor solution explained 59% of variation

Rogers et al. [19]	28-item empowerment scale	Principal components factor analysis	271 members of six self-help Programs	Five-factors: self-efficacy, power, community activism; righteous anger; and optimism and control over the future

**Table 2:** Results of exploratory factor analysis (EFA) of the PPES using principal component analysis with varimax rotation (*n* = 554).

PPES items	Factor loading			
	Factor 1	Factor 2	Factor 3	Factor 4
Item 1	0.82			
Item 2	0.83			
Item 3	0.84			
Item 6			0.79	
Item 7			0.85	
Item 10		0.88		
Item 11		0.88		
Item 12		0.67		
Item 14				0.58
Item 15				0.80
Item 17				0.74

% of variance	45.62	9.52	8.41	7.03
Cumulative variance	45.62	55.14	63.55	70.57
Cronbach's *α* of subscale	0.81	0.77	0.67	0.63

**Table 3:** Completely standardized solution for the second-order four-factor models (*n* = 554).

Variables	Estimates	Construct reliability*	Totally cumulative variances
Information	0.88	0.93	0.77
(1) The staff gave me clear information on how best to manage my illness.	0.84		
(2) Overall, I felt that I was talked at by the staff rather than listened to.	0.79		
(3) I wish I could have had more say in my treatment and care.	0.68		
Decision	0.89	0.80	0.72
(6) I felt that I always gave my consent before a clinical procedure was carried out.	0.78		
(7) I always felt that the purpose of my prescribed medication was fully explained.	0.65		
Individual	0.75	0.93	0.76
(10) The staff did everything possible to help me with anxieties over my illness.	0.84		
(11) The staff was always helpful and understanding over visiting times.	0.90		
(12) I felt that I was being treated as an individual by all members of staff.	0.55		
Self-management	0.84	0.84	0.62
(14) I had to ask for advice about what I should and should not do on discharge.	0.59		
(15) At no time did I feel that the truth about my condition was being hidden from me.	0.55		
(17) From time to time the staff gave me contradictory advice about my condition.	0.71		

*Estimate value greater than 0.50, construct reliability greater than 0.60, and totally cumulative variances higher than 0.50 were acceptable.

**Table 4:** Fit indices for the four-factor models and correlation between SPEQ and PSQ (*n* = 554).

Fit indices	Fit criteria	Four-factor model
Model *χ* ^2^ (df)	—	122.37 (40)
*P*	—	<0.001
*χ* ^2^/df (NC)	<5	3.05
Critical *N* (CN)	>200	281.15
RMSEA	0.05–1.0	0.061
IFI	>0.90	0.98
CFI	>0.90	0.98
NNFI	>0.90	0.98
GFI	>0.90	0.96
AGFI	>0.90	0.94
SRMR	<0.05	0.045

PPES four-factor	SPEQ	PSQ

Information	0.60**	0.56**
Decision	0.59**	0.48**
Individual	0.55**	0.60**
Self-management	0.47**	0.43**

PPES total score (11-item)	0.68**	0.64**

**P < 0.01.
